# Migrant (farm)workers and farmers in China and Myanmar: a perspective from the sugarcane sector

**DOI:** 10.1007/s10460-024-10701-0

**Published:** 2025-03-14

**Authors:** Chunyu Wang, Jingzhong Ye, Yiyuan Chen, Xiaobo Hua, Lu Pan, Yunan Xu, Jennifer C. Franco, Doi Ra, Sai Sam Kham, Saturnino M. Borras

**Affiliations:** 1 College of Humanities and Development Studies (COHD), China Agricultural University, No. 2 Yuanmingyuan West Road, Haidian District, Beijing, People’s Republic of China; 2 China and Global Development, Centre of Development Studies in the Department of Politics and International Studies (POLIS), The Old Schools, University of Cambridge, Trinity Lane, Cambridge, CB2 1TN UK; 3 Transnational Institute (TNI), De Wittenstraat 25, 1052 AK Amsterdam, The Netherlands; 4 International Institute of Social Studies (ISS) of Erasmus University Rotterdam, Korternaerkade 12, 2518 AX The Hague, The Netherlands

**Keywords:** Migrant farmworkers, Sugarcane, Labour regime, Land policies, Myanmar, Yunnan Province

## Abstract

In this paper we argue that the most critical factor that shapes the character and trajectory of the sugarcane sector in China is neither land nor labour, individually, but rather the interactions of social dynamics around land *and* labour, and specifically migrant labour. We argue that not only that the political economy of land and labour together drive agrarian transformation in the sugarcane sector, but more precisely that it is the process of how the labour regime shapes land politics, and how land politics shapes the labour regime, that is the central driving force. Furthermore, this mutual reshaping of land and labour regimes is multi-sited, occurring simultaneously within China and Myanmar, and in the China–Myanmar corridor. Our hunch is that the dynamics we observe here have broader resonance worldwide, especially in major farmer–farmworker land/labour flows along transnational corridors.

## Introduction

The agrarian transformation in China, as in many other countries, has been following a trajectory that is influenced by the state, capital, rural communities and nature, both domestically and globally. This means that the bigger picture of China’s agrarian changes is a complicated one, involving the emergence and persistence of a relatively modernized agriculture producing high-value agricultural goods based on household production, grafted onto and transforming land policy and politics in the process (Ho 2001; Ye 2015; Andreas and Zhan 2016; Zhan 2017). Philip Huang termed this “accumulation without proletarianization”, or “hidden agricultural revolution” (Huang et al. 2012; Huang 2016). It has been argued that this kind of agrarian transformation is possible because the land tenure system in China restricts large-scale land grabbing or land concentration (Zhang 2015; Zinda and Zhang 2017), and because the rapid industrialization and urbanization processes in China have attracted outflows of rural labour and reduced land–labour pressure (Huang 2016; Jacka 2018).

This type of analysis explains many aspects of agrarian transformation in China, mainly by focusing on the complex dynamics of land–labour regimes inside the country. But it is also criticized for failing to see the emergence of a “hidden” capitalist mode of production, in which hired labour plays a very important role (Yan and Chen 2015). There is no doubt that, in studying such economic and institutional shifts, land is an easier target than labour. That is true of China and many other regions where industrialization and urbanization occurred first and capital accumulation largely followed, during which rural labour flowed out of the villages in huge quantities and for successive years. These agrarian transformations are therefore generally characterized by the producers’ desperate pursuit of cheap (migrant) labour, for smallholders and for large farmers alike.

The sugarcane sector in China is a case in point. The sugarcane industry is still mainly household-based and heavily labour-dependent, relying particularly on cheap migrant labour from Myanmar and Vietnam. After four decades of development, production dropped dramatically from the early 2010s onwards (Wang and Xu 2022). To rescue the sugarcane industry from this dramatic collapse in production, the Chinese state and capital (represented chiefly by the sugar mills) have been taking a variety of economic, technological and institutional measures, aiming to improve or at least to stabilize production. In practice, these measures mainly comprise large land projects, facilitated by huge subsidies and changes in institutional or personnel arrangements. However, production has continued to fall in spite of all these efforts to entice or coerce farmers to engage in the sugarcane industry. The role of labour issues in this equation is reflected not in landless and jobless farmers and their movements, or in rebellions or silences (Wolford 2010; Moreda 2015; Hall et al. 2017), but rather in the absence of cheap migrant labour. Although the importance of cheap (migrant) labour to the agricultural production and food system has been much explored (Corrado et al. 2017; Pelek 2022), their importance to the survival of smallholders has not yet been much discussed, and let alone in the context where government interventions bring land dynamics into the picture.

We argue that the most critical factor shaping the character and trajectory of the sugarcane sector in China is neither land nor labour, individually, but the interactions of land *and* labour, and specifically migrant labour. In fact, we argue not only that land and labour together drive agrarian transformation in the sugarcane sector, but more precisely that it is the process of how the labour regime shapes land politics^1^, and how land politics shapes the labour regime^2^, that is the central driving force. Furthermore, this mutual reshaping of land and labour regimes is multi-sited, occurring simultaneously within China and Myanmar, and in the China–Myanmar corridor.

The remainder of the paper is organized as follows. A brief note on data collection methods is followed by a description of the declining sugarcane industry and the causes of its downturn. The following section then analyses the measures — old and new, economic, technological and institutional — taken by the state and the sugar mills to reboot the prosperity of the sugarcane industry. Finally, we explain how the sufficient supply of cheap labour contributes to survival and capital accumulation for both small and large farmers.

## Method

This paper is based on data collected from five fieldwork trips^3^ in ZK County, Lincang Municipality of Yunnan Province in China — two in 2019, two in 2023 and one in 2024 — and several field visits inside Myanmar.^4^

Yunnan is one of the two provinces in China in which sugarcane is produced on a commercially large scale (the other is Guangxi Province, where migrant labour from Vietnam plays an essential role). Yunnan shares borders with Myanmar, Laos and Vietnam. The sugarcane farmers in Yunnan have relied on cheap migrant Myanmar workers for harvesting since the 2000s (fieldnotes, April 9, 2023), at the same time that capitalists in China explore direct land investments in, and trade arrangements in the sugarcane sector with, Myanmar. The exploration of Myanmar as providing suitable land for sugarcane plantation became regularized in 2006 when a Memorandum of Understanding on Substitute Plantation was signed between Lincang Municipality and KoKang Region (China News 2006).

ZK County has four sugar companies, but one company, NTH Sugar Mill, stopped operation due to lack of adequate sugarcane to process in the 2022/2023 crushing season. Before 2020, there were usually more than 20,000 Myanmar workers in the county, arriving through both legal and illegal channels, mainly for cutting sugarcane. But the number dropped dramatically between 2020 and 2022 when the national border between China and Myanmar was closed due to the Covid-19 pandemic, with a wire mesh fence was set up by the Chinese government along its boundary.^5^

In the field visits, we went to four villages and two sugar mills in two townships. We interviewed the sugar mill managers (two), sugarcane sector programme staff in the Department of Agrarian Affairs (five), one labour contractor, seasonal Myanmar workers (fifteen), sugarcane farmers (twenty five) and village leaders (four) and village team leaders (four). We also interviewed two township leaders and the deputy head of the Bureau of Agriculture and Rural Affairs in the county. The findings and analysis presented here are based on these interviews and secondary data from the national and local statistical bureaus. This study is further informed by long-standing research on related issues by members of our team inside Myanmar.

### Declining sugarcane industry

Domestic sugarcane production in China has been declining over the past ten years. Sugarcane production and plantation areas reached their peak in 2013, recorded at 0.125 billion tons and 1,704,000 ha respectively for that year.^6^ Since then, both figures have been on a downward trajectory. According to national statistics for 2021, the plantation area had dropped to 1,316,000 ha and production had fallen to 0.114 billion tons.^7^

Zooming in on ZK County, the data show a very similar trend (see Table 1). In the 2022/2023 crushing season, a total of 153,700 mu^8^ of land were planted to sugarcane, less than half the figure for the peak period of 2012/2013 (340,000 mu).

**Table 1:** Sugarcane crushed in ZK County since 2012/2013

Crushing season	Area planted (in mu) in China	Area planted (in mu) in Myanmar	Cane from China crushed (in tons)	Cane from Myanmar crushed (in tons)
2012/2013	210,000	130,000	609,000	527,000
2013/2014	215,000	111,000	651,000	411,000
2014/2015	176,500	133,000	632,400	260,000
2015/2016	170,000	120,000	602,000	234,000
2016/2017	177,000	71,600	592,000	255,000
2017/2018	177,000	95,000	721,800	319,800
2018/2019	177,000	74,200	597,800	353,000
2019/2020	155,300	80,100	653,100	391,200
2020/2021	158,400	83,000	568,100	319,600
2021/2022	133,900	69,900	354,100	161,000
2022/2023	103,500	51,300		96,000

Data source: Bureau of Rural and Agricultural Affairs in the county

There are a number of important and converging reasons for the decline in production in ZK County and nationwide. First, China has experienced four decades of urbanization and industrialization, including a large-scale out-migration from rural to urban areas. This has left many rural villages in China without a sufficient supply of suitable agricultural labourers, because most of those who stayed behind are the elderly, children and those with health issues (Murphy 2002; Yan 2008; Ye and Pan 2011; Ye et al. 2013; van der Ploeg et al. 2014; Pan and Ye 2017; Ye 2018). This has resulted in the rising cost of agricultural labour, and the demand for migrant labour. Second, changing patterns in commodity markets have led to increased competition for land for other cash crops, such as coffee, tea, long beans, macadamia nuts etc. Third, the closing of borders during the pandemic resulted in a dramatic decrease of cross-border migrant workers. Fourth, since February 2021, large parcels of sugarcane plantations in Myanmar have been abandoned and/or left unharvested because of civil war. Fifth, the plots that are suitable for sugarcane plantation in China are scattered, small and located in sloping areas, making mechanization of production difficult if not impossible. The interweaving of these land and labour issues inside and outside China has been instrumental in the declining trend of sugarcane production.

## Increasing labour cost

**Fig. 1:**
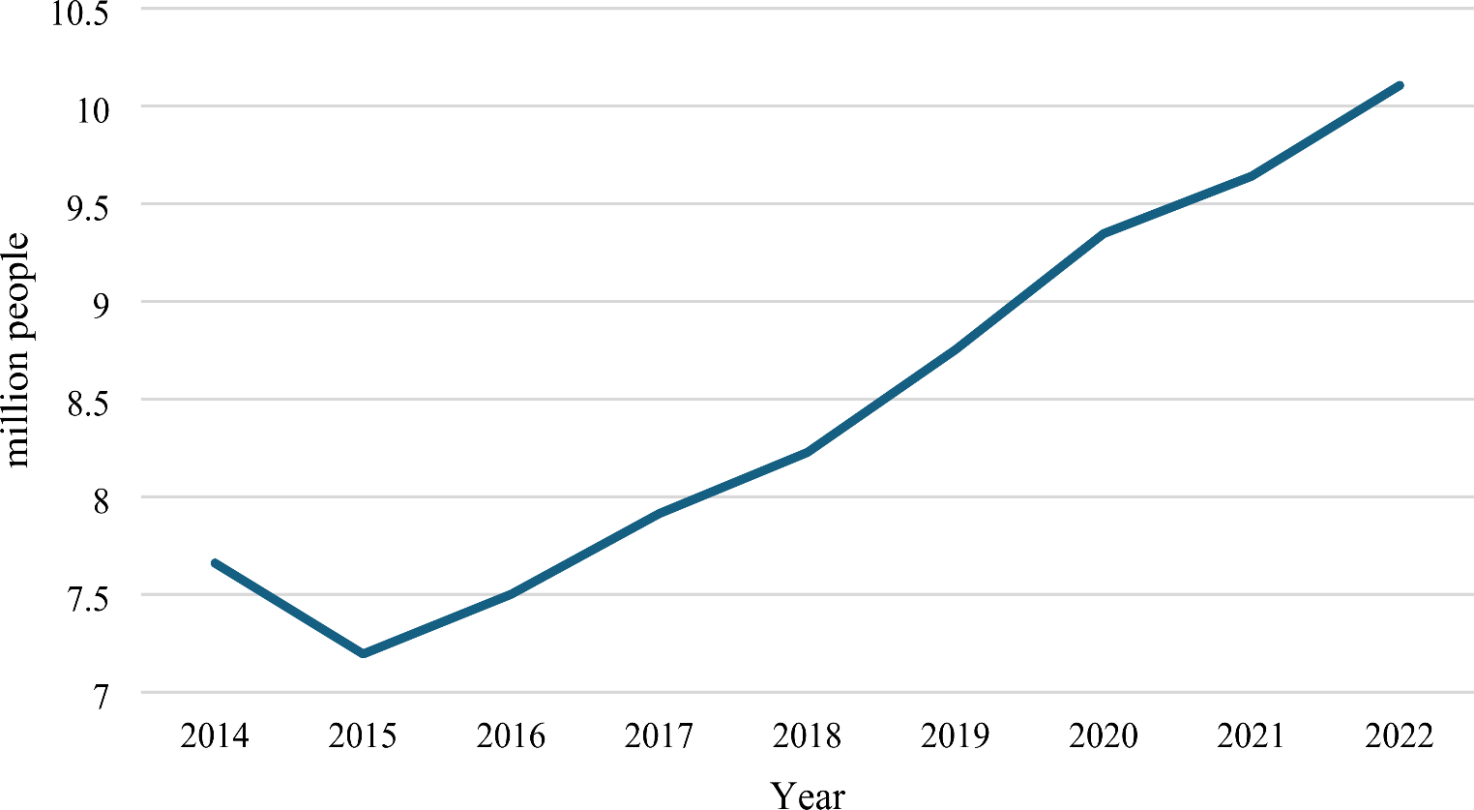
Migrant peasant workers in Yunnan Province (2014–2022). Data source: Statistical Bulletin of National Economic and Social Development of Yunnan Province. Data for earlier years not available

In China, the outflow of rural labour to urban areas, seasonally or permanently, started in the 1980s, and the total number of migrant ‘peasant workers’ (*nong ming gong*) reached 0.29 billion,^9^ accounting for more than half of the total 0.49 billion population living in rural areas, in 2022.^10^ In Yunnan, the number of migrant peasant workers was reported at 7.19 million in 2015, growing sharply to 10.1 million in 2022 (Fig. 1).

Corresponding with the sustained outflow of rural labour is the steady increase in the cost of wages for agricultural workers. In Yunnan, as pointed out by farmer Su and confirmed by other interviewees, the price for sugarcane cutting has risen continuously, regardless of whether the area planted with sugarcane expanded or shrank. In 2012/2013, the cutting price was 1.0–1.2 yuan/bundle,^11^ climbing to around 1.5–1.8 yuan/bundle in 2017/2018, around 2.0 yuan/bundle in 2019/2020 and 2.5–3.0 yuan/bundle in 2022/2023 (fieldnotes, July 6, 2023). The outflow of Chinese peasant workers has been somewhat offset by the inflow of migrant workers from Myanmar, which started in the 1990s (He 2008). The number of migrants working in ZK County before the pandemic is estimated at between 20,000 and 30,000 (fieldnotes, March 9, 2019), but the closed border and the border fence set up during the pandemic resulted in a dramatic fall in the number of Myanmar migrant workers, identified by local people as the main reason for the sharp increase in the cost of labour (fieldnotes, April 8, 2023; also Borras et al. 2022).

Labour costs were thus pushed upwards due to the shrinking supply of both domestic rural labour and cross-border migrant labour. Another factor at play in the supply of migrant labour from Myanmar is the fact that many of the Myanmar workers are farmers with access to land back in their home country. Some of them have crops such as tea and maize that require farmwork at the time of the sugarcane cutting season in China, which is usually after the Chinese Spring Festival. Many of these Myanmar farmers do not go to China at this time, resulting in higher labour costs for sugarcane cutting after the Spring Festival. The case of one large farmer in BK Village in Yunnan shows that the availability of domestic Chinese rural labourers is similarly dependent on the farming activities on their own lands. She reported happily that she was able to hire seventeen or eighteen Chinese workers this year because the spring came late and therefore the tea sprouted late. The local farmers chose to cut sugarcane for her first and then pick the tea on their own lands later. In years when the spring arrived at the normal time, she was unable to hire many Chinese workers (fieldnotes, April 7, 2023).

## Decreasing availability of land

Alongside the labour problems in the sector, there is also an issue with the declining availability of land. *Outside* China, farmland in Myanmar^12^ was largely not accessible during the pandemic, starting in late 2019, and during the civil war starting in February 2021. After the pandemic, some land became accessible again, but not all. Meanwhile, *inside* China, the competition for land from other crops which require less labour or have a higher market value is getting fiercer.

**Fig. 2:**
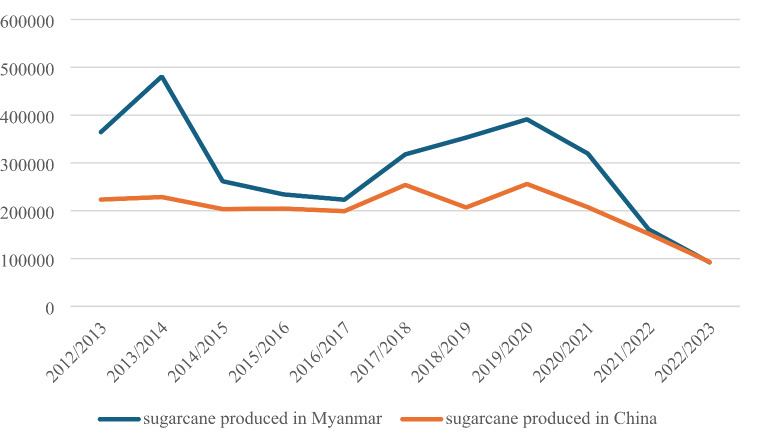
ZK County’s sugarcane production, domestically produced in China and imported from Myanmar (in tons). Data source: Department of Rural and Agricultural Affairs

A combination of these two factors led to a drop in production both for sugarcane produced domestically in China and for that brought from Myanmar and crushed in Chinese mills, for instance in 2012, 2015 and 2017, and from 2019 onwards. The decline of sugarcane production in China has been particularly evident since 2019 (Fig. 2).

Figure 2 shows that in the border county of ZK County in the majority of years, sugarcane imported from Myanmar and processed in China exceeded the volume of sugarcane produced and processed in China. Since 2021, the two sources of sugarcane have converged, both on a downward trend. As one interviewee, LZS, a sugar mill manager reflected, the MD Sugar Mill once handled more than 100,000 tons/year of sugarcane produced in and imported from three townships in Myanmar, but these were permanently lost due to the civil wars in 2015 and 2017 (fieldnotes, April 8, 2023). During the pandemic, the transportation of sugarcane from Myanmar also became more time-consuming and expensive with several rounds of inspections required, contributing further to the decline of sugarcane imports from Myanmar.

Inside China, the loss of land for sugarcane plantations is mainly due to increased competition from other crops. The farmers in ZK County used to plant maize and upland rice for subsistence farming, along with some cash crops, including sugarcane, coffee, tobacco, rubber, macadamia nuts, tangerines and (broad, long and French) beans. Tobacco is the traditional competitor for sugarcane; in 2022, tobacco brought 29.3 million yuan revenue for MD Township, almost three times that generated by sugarcane (fieldnotes, April 8, 2023). Beans are the new rising star, bringing a gross profit which ranges from 10,000 to 20,000 yuan per mu, depending on the market price. By comparison, the gross profit for sugarcane ranges from 2,150 to 3,440 yuan per mu (at 5 to 8 tons per mu, 430 yuan per ton). The beans are produced for supermarkets in big cities like Beijing and Shanghai. PMS Village, famous for its large-scale sugarcane production in the past, had more than 2,000 mu planted with different types of beans in 2022: “rich lands with access to water are all planted with beans now”. The total sugarcane plantation in PMS Village dropped from more than 10,000 mu in 2019 to around 7,000 mu in 2023 (fieldnotes, July 8, 2023). Some other crops are also popular because they require less labour and have local markets and processors, such as macadamia and tea.

## Difficult mechanization process

To produce sugarcane with fewer labourers and less land, China’s sugarcane industry has undergone government- and market-led transformations; the main objective of these transformations has been to mechanize and modernize the sugarcane industry. The Chinese government began to introduce foreign sugarcane machinery and production technologies in the 1970s, but the progress of research on machines suitable for the terrain in South China has been pretty slow. Mechanized ploughing, sowing and harvesting was carried out on 86.42%, 61.91% and 66.56% of China’s arable land, respectively, in 2022, with the comprehensive mechanization rate of crops reaching 73.11% (Ministry of Agriculture and Rural Affairs of PRC 2024), but the comprehensive mechanization rate of China’s sugarcane industry was much lower than the average level (around 60%), and the mechanized harvesting rate was only about 4% (Luo 2023).

The challenges in advancing the mechanization and modernization of the sugarcane industry have been multiple, including but not limited to complex natural conditions and unsuitable machinery design. For example, the large body of a standard combine harvester cannot operate in the patchwork of small plots typical in China; the ‘whole-stem combine’ harvester is unable to harvest fallen sugarcane; and the ‘leaf-stripping technology’ is not mature; more importantly, the overall agronomic conditions in China’s sugarcane areas are not up to the requirements of mechanization, given narrow row spacing, the type of ploughing used, and the narrow strips of land on the hills. An informant summarized it for us as follows in an interview: “cutting sugarcane with machines wastes too much sugarcane and leaves too many unwanted leaves and tips”; and “machines ruin the roots and the field, and the tillering in the next year will be damaged” (fieldnotes, July 8, 2023). While there are admirable innovations in the sugarcane sector in China, such as the terracing campaign (converting sloping hills into terraced land to allow for sugarcane production) and the invention of narrower and lighter combine harvester machines, these are still not significant enough to offset the large-scale need for agricultural labour from the kind of wage workers that have traiditionally come from Myanmar.

## Land projects and related mechanisms

Some old and some new economic, technological and institutional measures have been initiated and disseminated by the central state and the sugar mills in order to improve or at least maintain sugarcane production at current levels. The three most prominent measures are: (1) terracing projects to improve the land, and enable mechanized farming; (2) the establishment of agricultural service companies for mechanized production and labour contracting, such as combine harvesting and pesticide spraying by drone companies (Chen and Jiao 2024); and (3) special institutional and personnel arrangements in the local government and the sugar mills to entice or coerce farmers to engage in sugarcane production. These are relatively recent innovations. Whether or not they will reverse the downward trend in sugarcane production, or even boost production to new higher levels, it is too early to say. We will explain each of these measures briefly.

### Large-scale terracing projects

The main purposes of terracing projects are to improve the average productivity of the land, expand the sugarcane plantation area, and reduce the reliance on labour by enabling greater usage of machines.

It is difficult to trace exactly when and where terracing projects started in China.^13^ Sporadic terracing projects in ZK County began in 2017, while large-scale terracing projects accompanied with increased mechanization were initiated in 2020 and can be divided into two phases. The first round of terracing (2020–2021) involved a total investment of 30 million yuan (20 million from the state fund and 10 million from the sugar mills). In that phase, 30,000 mu of arable land was regained for sugarcane plantation. The second round of terracing (2022–2023) involved an investment of 40 million yuan in total (50% from the state government and 50% from the sugar mills). In the second phase, terracing projects created approximately 20,000 mu of farmland and built road and other infrastructures necessary for sugarcane plantation and transportation (fieldnotes, April 8, 2023).

The precondition for the sugar mills to make such large investments is that the farmers should commit to plant sugarcane for at least three years on the terraced land. A subsidy of 350 yuan (almost $50) per mu is transferred to the farmers as long as they plant sugarcane in the newly terraced land. If terraced land is not planted with sugarcane, the farmer will be charged a fine; in BK Village, for instance, the fine is around 200 yuan (lump sum) (fieldnotes, July 9, 2023). As one clerk^14^ in charge of sugarcane production in the sugar mill remarked, the majority of terraced land has been planted with sugarcane. For example, between 2020 and 2023, PT Village created 1,800 mu of terraced land, of which only 80 mu has been planted with maize and non-irrigated rice rather than sugarcane. The owner of these 80 mu of maize and non-irrigated rice claimed they were too old to plant sugarcane (fieldnotes, July 8, 2023).

However, it is too early to say whether the main objectives of this land project (to reduce the reliance on labour and improve mechanized production) have been met. Initial data show that terracing is usually undertaken on sloping areas where the land was originally either idle or planted with fast-growing trees, maize or non-irrigated rice, whose owners have little interest in engaging in sugarcane production. After terracing, an important task for clerks in the sugar mill is to persuade the original owners of the lands, or other farmers, to plant sugarcane. These newcomers may be recruited from either inside or outside the village. They usually rent more than 100 mu of land from several small households so that they are able to make enough profit (fieldnotes, April 6, 2023). This results in an increase in the average land size held by each individual sugarcane farmer, which also increases the need to hire more labour to till their land. In this regard, the reliance of the sugarcane industry on labour remains, or even grows due to the increased average farm size after the terracing project.

## Mechanization

Terracing improves the productivity of land by stabilizing the soil and retaining the moisture, and making the land more accessible to some small agricultural machines. However, the mechanization level in sugarcane production remains low. The terracing project has been accompanied by the promotion of mechanization through huge subsidies from the central government: 170 yuan per mu for mechanized ploughing; 20 yuan per mu for unmanned aerial vehicle (drone) pesticide spraying (with another 15 yuan from the sugar mill); 45 yuan per ton for mechanized harvesting; and 20 yuan per mu for crushing sugarcane leaves and returning them to the field. Sugarcane farmers in ZK County usually take the first two or three types of subsidies mentioned above — i.e., 350 yuan for newly planted sugarcane, 170 yuan for mechanized ploughing and 20 yuan for drone pesticide spraying (a total of 540 Yuan/mu in subsidies) — but reject the subsidies for mechanized harvesting and smashing sugarcane leaves, because the costs of these two activities far exceed the subsidies provided (fieldnotes, July 8, 2023).^15^

In ZK County, mechanized production is mainly provided by one agricultural service company (HM Agricultural Service Company) and four farmers’ cooperatives. This company has the capacity to mobilize large and micro tractors, spreaders, drones, grippers etc. inside and outside the county. It has three teams — one for drones, one for tractors and a third one for infrastructure (including the road building which is necessary for transporting sugarcane). Farmers are charged 730 yuan/mu for mechanized ploughing (of which 350 yuan per mu is for using the machine and 380 yuan per mu is for labour), and 255 yuan/mu for field management (of which 100 yuan is paid by the sugar mill, 25 yuan is paid by the government as a subsidy to farmers, and the remaining 130 yuan is paid by the farmers themselves). Mr. Zhang, the operating manager and the head of Team One, reported that his team provided agricultural services to around 200 farmers, including six large farmers, amounting to a total of 4,000 mu in 2022. Separately, the company also provided agricultural services to 900 poor farmers in ZK County (a total of 20,000 mu), subsidized by a poverty alleviation project (fieldnotes, April 10, 2023).

Apart from the subsidies offered by the government, the sugar mill has long been providing financial support to any sugarcane farmer in need. Sugarcane farmers can easily get loans from the sugar mill for land rent and labour hire, and for agricultural inputs such as sugarcane seeds, plastic film, fertilizers, chemicals etc., which are paid back after the sugarcane has been harvested. The maximum payment in advance from the sugar mill amounts to 1,760 yuan per mu (450 yuan/mu for seedling, 400 yuan for fertilizers, 20 yuan for chemicals, 160 yuan for plastic film, 350 yuan for semi-mechanized planting, 380 yuan for labour service).

Whether or not the goals of mechanization are ultimately met, these subsidies and loans demonstrate the commitment of the state and the sugar mill to the mechanization process; together, they have invested heavily to ensure that farmers, large and small alike, are able to engage in the sugarcane industry without being hindered by lack of capital.^16^

## Special institutional and economic arrangements

With sugarcane being one of the pillar industries in ZK County (as the main source of revenue, only recently exceeded by tobacco; field notes, April 8, 2023), the local government and the sugar mill have created a personnel structure aimed at encouraging farmers to farm sugarcane, and to do so in the ways that they prefer.

At local government level, a special working team has been established in each township. In MD Township, for instance, this working team consists of five government officials in charge of promoting sugarcane production in ten villages in the township. Mr. Lee, the official assigned to PMS Village and BK Village, explained that he is supposed to visit these two villages every week, to communicate with village leaders about field management (and the progress of terracing projects in recent years). He also contacts government technicians when necessary. There are also some additional subsidies to encourage sugarcane farmers to spend more time in the field, such as 25 yuan/mu/year for middle-term management and 100 yuan/mu for using plastic film to cover the roots after harvesting (fieldnotes, July 8, 2023).

**Fig. 3:**
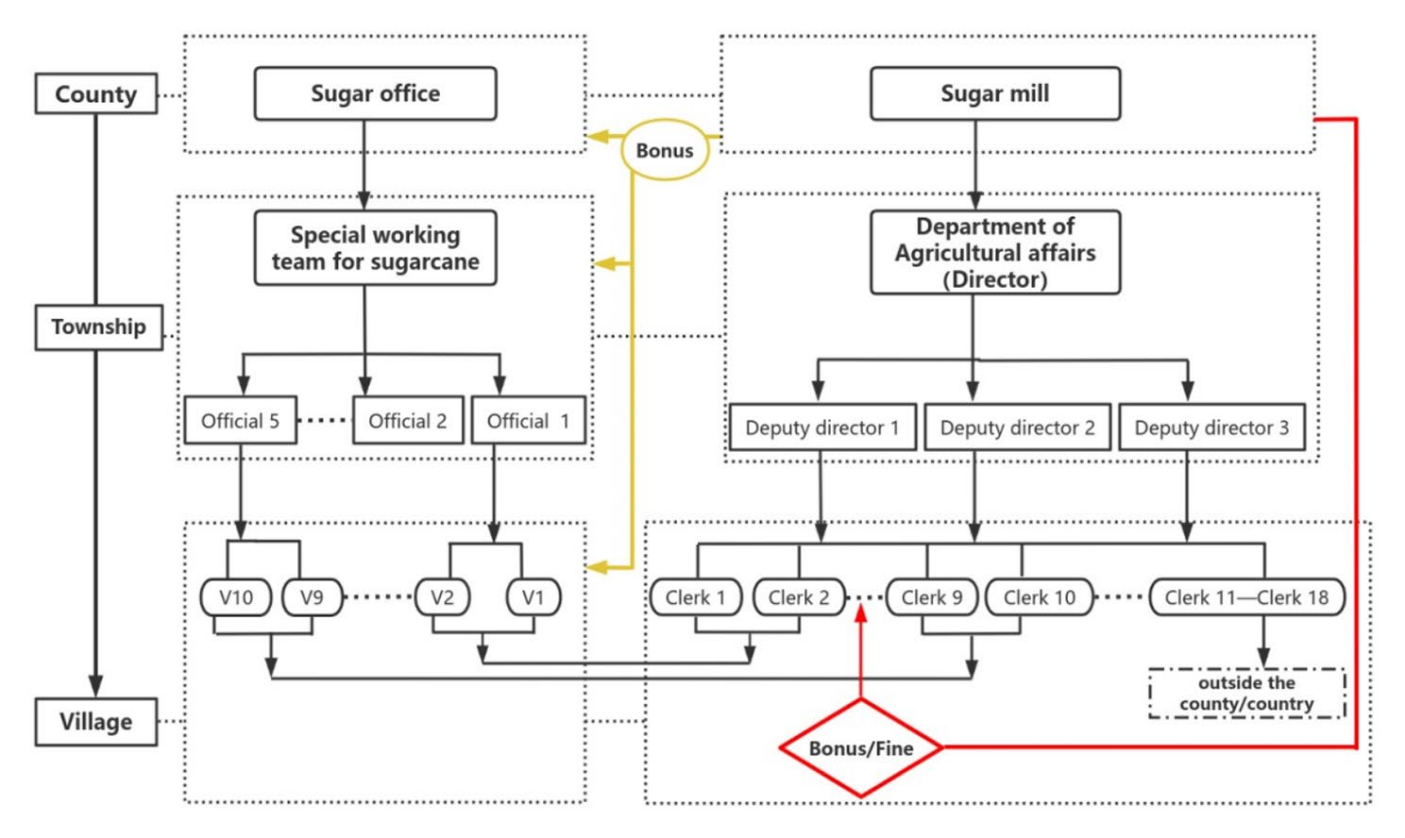
Personnel arrangements of the government (lefthand panel) and the sugar mill (righthand panel) for production. **Note** Due to limited space, the figure does not list out all officials (five), clerks (eighteen) and villages (ten). V stands for village

For its part, the sugar mill set up the Department of Agricultural Affairs even before the mill had started operating. In the sugar mill in MD Township, there is one department head, three deputy heads and eighteen clerks in charge of eighteen sugarcane zones in and outside China. For each sugarcane zone, the clerk is assigned the task of expanding the sugarcane plantation annually, according to the estimation of land that is available and suitable for sugarcane. To fulfil the task, the clerk recruits an assistant in the administrative village and appoints several sub-team leaders in the corresponding natural villages in the administrative village. “Their daily work is to visit and persuade the farmers to plant sugarcane or to improve the field management” (fieldnotes, July 9, 2023). Figure 3 shows how the officials and the clerks work together to promote sugarcane production.

The system also incorporates a punishment and bonus system in relation to each administrative level. The sugar mill provides a yearly bonus to the county government (2 yuan per ton), the township government (1.5 yuan per ton) and the village committee (1 yuan per ton) for sugarcane production. For the clerk in charge of a specific sugarcane zone, an extra bonus is given if the goal of sugarcane expansion is met, but a fine is imposed if this task is not accomplished.

For example, Mr. Kui, responsible for BK Village, was allotted the task of increasing his sugarcane area by 1,500 mu in the 2023/2024 crushing season. If he succeeded in adding 1,600 mu of newly planted sugarcane, he would receive a bonus of 24,500 yuan — 15 yuan per mu for fulfilling the original task and 20 yuan per mu for his extra achievement (15 yuan * 1500 mu + 20 yuan * 100 mu). But if he failed to reach this target with, for instance, only 1,300 mu of newly planted sugarcane (200 mu less than the target), he would be fined 1,000 yuan, 5 yuan per mu for the unfinished task (5 yuan * 200 mu).

Mr. Kui’s assistant also gets paid by the sugar mill at a rate of 650 yuan per month (for 10 months per year), plus 0.2 yuan per ton produced by the whole village in a year. The sub-team leaders do not have salaries, but do get a bonus from the sugar mill of 0.1 yuan per ton produced by the natural village of the sub-team leader. Mr. Kui was appointed as the clerk responsible for BK Village in 2017; since then he has fired two assistants and four sub-team leaders who were not doing the job well, to make sure he could achieve his target in every crushing season. Of the eighteen clerks in the sugar mill in charge of sugarcane production, only two reached their targets; Mr. Kui is one of them (fieldnotes, July 6, 2023).^17^ The reasons for his success will be explored further below.

Apart from all these efforts, the local government and the sugar mill adopted two additional measures to solve the problem of labour shortage during and after the pandemic. One is to encourage “mutual aid” teams at the village level. In this scheme, families which have access to less labour and therefore cut less sugarcane, have to pay their neighbours/friends for any extra bundles they help to cut, at the market price of 2.5–3.0 yuan/bundle. This is actually an organized labour market to mobilize and ensure the supply of labour, not “mutual aid” in the normal sense. This rate is much higher than that paid to migrant Myanmar workers before the pandemic, which was 1.5–1.8 yuan per bundle, because Chinese people are unwilling to cut canes for such a low rate. The other measure is to resort to labour contractors/companies. In ZK County, HM Agricultural Service Company also connects with other labour-contracting companies to bring extra labour into local agricultural industries. In the 2022/2023 crushing season, it managed to recruit two teams with about forty workers each from Myanmar, but the majority of labour (about 600 Chinese workers) came from adjacent poorer counties such as BS County. “Hopefully we could recruit more from Myanmar later this year [2023] when polices are relaxed and border gates reopen in Myanmar”, said manager Zhang of HM company (fieldnotes, April 8, 2023). Both measures can bring more labour into the field, but not *cheap* labour.

Our findings demonstrate that the agrarian transformation in the sugarcane industry, led by the state and the sugar mill, is clearly mechanization-oriented and technology-guided, supported by large projects, huge funds and well-designed personnel arrangements. The issues of land productivity and capital have been the central concerns of these efforts. However, as noted earlier, the labour question remains unresolved.^18^

### Cheap labour as key to sugarcane production

The availability of cheap labour in sugarcane production is important in three main ways. First, it impacts on the ability of large farmers to expand, and of small farmers to survive. According to the calculation of some large farmers in Guangxi Autonomous Region, labour accounts for the largest proportion of total costs, at around 39.1%, compared to other costs like land rent (18.6%), agricultural inputs (27.9%) and machinery (14.4%) (Wu 2020: 145; see also Xu 2020 for broader context). Our calculation for Yunnan suggests that the share of labour costs has become even more prominent: Table 2 shows that labour costs amount to an average of 47.5% of the total costs for large farmers in Yunnan. (Subsidies discussed in the previous section are shown in Table 2 as deductions.)

**Table 2:** Sugarcane production cost for large farmers (over 100 mu), in yuan/mu

Cost	Items	Year 1	Year 2	Year 3	Average over 3 years
Land	Land rent	150	150	150	150
	% in total cost	6.7%	11.4%	11.4%	9.8%
Agricultural inputs	Seeds	450	0	0	
	Fertilizers	400	350	350	
	Plastic films	0	160 − 100 = 150	200 − 100 = 100	
	Chemicals	20	20	20	
	Sub-total	870	520	520	635.7
	% in total cost	38.8%	39.3%	39.3%	39.1%
Machines	Ploughing	320 − 170 = 150	0	0	
	Drones	60-25-15 = 20	60-25-15 = 20	60-25-15 = 20	
	Sub-total	170	20	20	70
	% in total cost	7.6%	1.5%	1.5%	3.5%
Labour	Planting	380	0	0	
	Cutting	500	500	500	
	Middle-term field management and others	130	130	130	
	Sub-total	1050	630	630	770
	% in total cost	46.9%	47.8%	47.8%	47.5%

Data source: estimated by three large farmers and compiled by the authors (fieldnotes, April and July, 2023)

Note: subsidies are shown as deductions from costs

As for small farmers, household labour is able to reduce the burden of paying for external workers, but only to a certain extent; usually the labour of only two or three household members could be mobilized. Small farmers also need to hire-in labour to cut canes or undertake some field management tasks (fieldnotes, July 9, 2023). As the cutting price increased from 1.0 to 1.2 yuan/bundle in the 2012/2013 crushing season to 2.5–3.0 yuan/bundle in 2022/2023, meanwhile the sugarcane price remained at 420 yuan per ton for 10 years^19^, the net profit of producing sugarcane dropped from 850 yuan per mu to less than 500 yuan per mu. When hiring-in workers from Myanmar, the daily cost is 85 yuan per day per labourer, whereas for Chinese labour the cost is 120–130 yuan per day (fieldnotes, July 7, 2023). A lot of small farmers have been squeezed out of sugarcane production during these years because the total net income from their small plots could no longer meet their needs of their family members, dropping from 25,500 yuan/year ten years ago to less than 15,000 yuan/year (without adjustment in inflations), given 30 mu of land for a small sugarcane farmer (see also Zhang 2019). An additional note for the drop-out of small farmers in sugarcane industry is that it is getting more difficult for them to hire in labour in comparison to large sugarcane farmers, mainly because small sugarcane farmers couldn’t offer enough odd jobs to these migrant labour and these labour prefer to stay with large farmers. “(small farmers have) only a few mu of sugarcane… and after cutting those down we have to move to look for other jobs. That’s too troublesome” said HS, a migrant worker in ZW village (fieldnotes, March 9, 2024). In BK village for instance, cheap migrant labour is relatively abundant (more discussions about this village are below), but the number of small sugarcane farmers still dropped rapidly. There were 164 sugarcane farmer in 2012/2013 crushing season, out of which 140 farmers planted less than 30 mu. But the total number of sugarcane farmers decreased to 100 in 2022/2023 crushing season, out of which only 24 farmers planted less than 30 mu (fieldnotes, January 13, 2024). These small sugarcane farmers either rented out their land to large farmers and outmigrated for other types of livelihoods, or shifted to other crops like beans, oranges, and macademia trees (fieldnotes, March 9, 2024).

The second key role of cheap labour in the sugarcane industry is reflected in the success or failure of expanding sugarcane plantation through terracing projects. The outstanding performance of Mr. Kui in BK village cannot be attributed to his personal capabilities alone. Firing assistants and sub-team leaders who didn’t work hard enough is apparently not the full answer to his success, as other clerks also fired assistants for better performance, according to the clerk in charge of PD Village (fieldnotes, July 7, 2023), but without achieving the same results as Mr. Kui.

The sugarcane plantation area in BK village increased from 1,800 mu in 2020 to 4,600 mu in 2021. In 2022, an additional 1,300 mu of sugarcane was planted, and in 2023, another 1,600 mu was added, making a total of 7,5000 mu of sugarcane plantation in BK Village. At the time of the fieldwork, there were ten local large sugarcane farmers, four large farmers from other villages, and three Myanmar large farmers (fieldnotes April 8, 2023). Such a large expansion of sugarcane plantation is possible because of the successful implementation of terracing projects, enabling Mr. Kui to persuade farmers into planting sugarcane. Mr. Kui succeeded in his persuasion, mainly because BK Village is, in general, not short of cheap labour. This village is along the China–Myanmar border, and at the time that civil war broke out in Myanmar more than ten years ago, it accommodated around 360 Myanmar workers (excluding 110 Myanmar brides who married villagers). Ten Myanmar families (less than 20% of the total migrant Myanmar families) in this village got the newly terraced land and started to plant sugarcanes. Three of these families became large sugarcane farmers in China due to the terracing project.

The three cases below (two in BK Village and one in ZW Village) clearly show the link between local farmers’ willingness to engage in the sugarcane industry and the availability of cheap labour.

#### Case 1

*LWM*,* a female farmer*,* 34 years old*,* has 200 mu of sugarcane and over 100 cows in her family. She also has a primary tea factory. During the Myanmar civil war*,* the family offered shelter to about 14 households from Myanmar. These households live in her cattle farm. There are at least 12 workers in these households. She has no worries about shortage of labour. She entered into the sugar industry in 2021/2022*,* and produced 770 and 800 tons in 2022 and 2023.* (BK Village, fieldnotes, April 7, 2023)

#### Case 2

*HYL*,* a female farmer*,* 32 years old*,* has around 250 mu of sugarcane and 30 mu of tea. She gave the latter to three Myanmar families; each has 10 mu. She also accommodated 18 Myanmar workers who came to China more than 10 years ago*,* among whom there are five kids. Some kids are about 11 to 12 years old*,* “old enough to cut sugarcane”. She also has no worries about shortage of labour and produced 700 tons in the last two years because her land is steeper*. (BK Village, fieldnotes, April 7, 2023)

#### Case 3

*HZL*,* a male farmer*,* 54 years old*,* had 17 mu of sugarcane in 2012*,* 50 mu in 2013*,* 100 mu in 2020*,* and another 100 mu in 2021*,* now he has more than 250 mu of sugarcane. In 2015*,* he accommodated two Myanmar refugee households. Gradually*,* he accommodated nine households. “You need to have workers and then you could rent more land (to farm)”.* (ZW Village, fieldnotes, July 8, 2023)

PT Village offers a good case in comparison to BK Village. PT Village is also a border village. Before the pandemic, Myanmar workers usually came through the mountains, with numbers reaching as high as 1,000. But during and after the pandemic, this (illegal) inflow of migrant workers stopped due to the construction of border fences, and the village accommodated only twelve households of Myanmar refugees (ninety-one people including forty-six workers).^20^ The land used for sugarcane production decreased sharply from over 6,000 mu before the pandemic to only 4,420 mu in 2023 (fieldnotes, April 8, 2023).

The third important role of cheap labour in sugarcane production is demonstrated by the popularity of “jump-the-gun cutting” and “extra-cutting”. These phenomena occur due to the shortage of cheap labour. “Jump-the-gun” cutting implies that the timing of sugarcane cutting is determined by the ability of farmers to hire in migrant agricultural workers. “Extra-cutting” refers to farmers cutting more cane than the sugar mill allows per day. Cutting is conducted according to the availability of cheap labour, and not according to cutting tickets issued by the sugar mill.^21^ Although the sugar mill has imposed fines on these farmers and corresponding mill clerks, this unauthorized cutting is still popular in the region. This reflects, once again, the importance of cheap and available labour to the sugarcane industry, and weakens the control of the sugar mill over farmers’ harvesting behaviour.

## Conclusions

This paper investigated the decline of sugarcane production in China and the land projects and measures undertaken by the state and capital (in this case the sugar mills) to reverse the trend. We studied this phenomenon by looking at the role played by land and labour dynamics inside and outside China. Our main finding is that the most important factor behind the relative decline in the sugarcane industry in China is neither land nor labour, individually, but rather the interaction of the political economy of land and (migrant) labour in the sector. The trajectory of change has been impacted less by the parallel existence of land and migrant labour; rather, it is the process in which labour regimes have shaped land dynamics, and land policies have shaped labour regimes that matters most.

### Land policies shaped by labour regimes

The labour regime shapes land policies in two ways. First, the large-scale terracing project in China is an immediate response to the absence of cheap migrant labour from Myanmar. It started in 2020, at the very beginning of the pandemic, when the border fence and closed border crossings made both legal and illegal migration almost impossible. This project, together with other institutional and economic arrangements, is intended to reduce the reliance of the sugarcane industry on labour by introducing machines into the field, and at the same time to entice or coerce farmers to plant sugarcane. Mechanization is difficult in the institutional setting of China in general, and in the geographical conditions in South China in particular. Considering these difficulties, the effort to push mechanization in China through land projects is best understood through the lens of shortage of labour, particularly cheap migrant labour.

Second, and perhaps more significantly, farmlands originally allocated only to the Chinese farmers are now open to Myanmar refugees. The state and the sugar mill, desperate for farmers to work on the newly terraced land, could not find enough Chinese villagers to plant sugarcane; they therefore recruited Myanmar refugees to take over the newly terraced lands to farm sugarcane. This is evidenced by the fact that villages with more Myanmar refugees (like BK and ZW villages) were able to successfully expand the sugarcane plantation areas while in villages with fewer or no Myanmar refugees (such as PT and PMS villages) the sugarcane plantation areas are shrinking. In this way, land projects can become a social differentiation tool for migrant Myanmar refugees. A few Myanmar refugees (less than 20% of the population in some villages) become sugarcane farmers in China. Many are captured as agricultural workers in the villages.

### Labour regimes influenced by land dynamics

The land dynamics in Myanmar and the land projects in China have two important impacts on the labour regimes in the two countries. First, land back home (for Myanmar farmers) influences the availability of rural labour in the sugarcane industry (Borras et al. 2020; Ra et al. 2021; Kham 2023). In previous years, many migrant Myanmar workers went back home to prepare their own land during February and March — a time when Chinese farmers are also busy with their own plots. The resulting shortage of Myanmar workers in the labour market pushed the cutting price higher during those months. Since then, civil wars in Myanmar from the 2000s onwards, but particularly since the military coup in early 2021, have deprived many Myanmar farmers of their land and homestay, forcing some of them to flow into China and become farmers or agricultural workers there. That the migrant labour regime shapes land policy dynamics was also the conclusion of Hua and colleagues in relation to upland southern China (Hua et al. 2019; Wang and He 2024) and of Xu (2020) in the context of Guangxi. Second, Chinese sugar mills lost access to sugarcane farmland in Myanmar during the civil wars; this undoubtedly pushed these sugar mills to look for more land inside China.

However, land projects in China might have unexpected consequences on the labour regime. These projects, originally intended to increase mechanization and reduce labour requirements, seem to be resulting in even heavier reliance on cheap migrant labour. After terracing, the production unit becomes larger, and current agricultural machinery is not suitable for the narrow strips of land that have been created. Family labour and exchange of labour are not adequate either, which means that hired labour is proving to be essential in these newly terraced lands.

### Cheap migrant labour and capital accumulation of small and large farmers

The third important finding in this paper is that cheap migrant labour is vital for the survival of small sugarcane farmers and the expansion of large sugarcane farms. This can be inferred from the fact that a majority of small sugarcane farmers have had to quit sugarcane production since 2020 because of a lack of cheap migrant labour. Without cheap migrant labour, these small farmers cannot survive on planting sugarcane. It can also be inferred from the fact that some border villages which house Myanmar refugees who can be used as cheap (and captive) labour, have been able to expand their sugarcane plantations successfully, and large farmers are willing to invest in this industry. We believe that the availability of cheap labour could explain the capital accumulation of (large and some small) sugarcane farmers in China, and could also explain the survival of smallholders in Europe who use refugees from Africa and the Middle East to provide cheap labour (Erturk 2017; Pelek 2022). This reality is also found outside the sugarcane sector in southern China, as Zinda and He (2020) and Hua et al. (2023) have shown.

Land and labour dynamics inside and outside one country offers a useful lens through which to understand agrarian transformations in China and beyond. In some economic and institutional settings, land is relatively easy to secure (for instance, land abandoned due to outmigration, under a powerful authority and with huge subsidies) but labour is not. Agrarian transformations in these settings are characterized by the challenging pursuit of cheap migrant labour, reflecting how important such labour is to the survival and capital accumulation of both small and large farmers. By exploring this phenomenon, this paper speaks to the cluster of literature in Critical Agrarian Studies (Edelman and Wolford 2017; Akram-Lodhi et al. 2021; Borras 2023) on the relationship between land and labour, specifically migrant labour. It identifies with research on the trend towards diversification of livelihood sources of working people, both land-based and wage work, as articulated in Bernstein’s notion of “classes of labour” (Bernstein 2010), as well as the important elaborations of this idea by Pattenden (2018), Cousins et al. (2018), and Shah and Lerche (2020), and the reconceptualization of “working people” by Shivji (2017). It also resonates with the argument of Borras and Franco (2023) that we need to focus on the interrelations among farmers, farmworkers and other working people in agrarian and rural areas and across the rural–urban corridor. We believe that the social dynamics we observed in our case study have broader resonance worldwide, especially in major farmer–farmworker transnational corridors, such as Nicaragua–Costa Rica, Mexico–United States, Brazil–Paraguay, Zimbabwe–South Africa, and Africa–Europe.

## Footnotes

1 By land politics, we refer to political contestation around access to and control over land in the context of land in production/social reproduction connected whole (Borras and Franco 2024a). The terrain for contestation has been recently reshaped by the global land rush that in turn influenced the extent and trajectory of labour mobility within and across sectors and countries (Borras and Franco 2024; Wolford et al. 2024).

2 By labour regime, we “refer broadly to different methods of recruiting labour and their connections with how labour is organized in production (labour processes) and how it secures its subsistence” (Bernstein 2010).

3 The research team in the field in China includes: Jun Borras, Jingzhong Ye, Chunyu Wang, Lu Pan, Yiyuan Chen, Xiaobo Hua, Doi Ra, Sai Sam Kham, Yanglin Liu, Xiaohui Shi, Jennifer C. Franco, Kamoon, and Phye Phyu Thet Htway.

4 The research team in the field inside Myanmar includes: Doi Ra, Sai Sam Kham, Jennifer C. Franco and Jun Borras.

5 See video on *How fences are set up*. Tencent Net. 2023. https://v.qq.com/x/page/p3259t07wze.html. Accessed 14 September 2023.

6 National Statistical Bureau. 2023. http://www.stats.gov.cn/sj/ndsj/2022/indexch.htm. Accessed 25 July 2023.

7 National Statistical Bureau. 2023. http://www.stats.gov.cn/sj/ndsj/2022/indexch.htm. Accessed 25 July 2023.

8 Fifteen mu equals to one hectare.

9 National Statistical Bureau. 2023. Report on Migrant peasant workers 2022. https://www.gov.cn/lianbo/2023-04/28/content_5753682.htm. Accessed 14 September 2023.

10 National Statistical Bureau of China. 2023. China Statistical Yearbook. http://www.stats.gov.cn/sj/ndsj/2022/indexch.htm, Accessed 14September 2023.

11 In Yunnan Province, each bundle weighs about 35 kg.

12 Farmland outside China has not been restricted to sugarcane, nor to Myanmar. See Hua et al. 2022.

13 The first academic paper about terracing projects in China was published in 1979, discussing how to calculate the area of the land regained through terracing. However, this agricultural practice has a tradition of several centuries in China.

14 The role of the clerks is described in more detail below.

15 These agricultural practices are checked carefully by the clerks in the sugar mill in each plot, and the clerks send lists for subsidies to the local government. The local government will then inform the rural credit bank to transfer the appropriate subsidies directly to each farmer’s bank account.

16 This is also a way of persuading (or capturing) the farmers to engage in sugarcane production.

17 The other clerk who succeeded in meeting his target is responsible for a border village outside the county. Unfortunately, we were unable to interview him during our field trip.

18 There are some labour contractors trying to bring migrant Myanmar workers to ZK County, but the numbers are very small due to the restrictions on cross-border migration imposed by the central state since the pandemic. During our field trip in April 2023, a migrant worker team-leader reported that he had thirty-six migrant workers in his team (five families including twelve kids) — far less than the eighty to ninety workers he had before the pandemic. This is mainly because the border crossing in Myanmar which is the counterpart to *Qingshuihe* Gate in Yunnan Province, China, is still closed.

19 The sugarcane price rose to 450 yuan per ton in 2023/2024 crushing season (fieldnotes, January 13, 2024). That makes the net profit of producing sugarcane reach at approximately 620 yuan per mu.

20 Refugees from Myanmar share the same languages, the same religions and even blood ties with local villagers in China. They congregate mainly in villages along the China–Myanmar border, such as BK Village. Their labour situation and social differentiation are worthy of a separate paper, but fall outside the scope of this article.

21 This is a system set up by the sugar mills to control the quantity of raw materials sent to the factory every day. A ticket is a cane-cutting certificate with the name of the farmer, the number of canes cut, the date they are sent to the factory, and the number of the dispatching order. Only with this certificate can the cut sugarcane be unloaded into the factory by the vehicle arranged by the sugar mill. Sugar mills set the number of cutting tickets according to the production capacity of the mills and issue tickets according to the distance and maturity of the cane. Cane tickets are used to coordinate the harvesting and transportation of sugarcane within the cane area and to reduce the loss of sugarcane due to uncontrolled harvesting.
